# “We do what needs to be done”: caregivers’ experiences of healthcare and support for people with multiple long-term conditions in the last year of life

**DOI:** 10.1186/s12904-026-02115-y

**Published:** 2026-05-27

**Authors:** Sarah P Bowers, Ellen Kerr, Eilish Murphy, Jillian Galbraith, Colin McIntyre, Margaret C Weir, Maureen Ward, Sarah E E Mills, Linda Williams, Jo Bowden, Frances Quirk

**Affiliations:** 1 School of Medicine, University of St Andrews, North Haugh Street St Andrews, KY16 9TF UK; 2 NHS Fife, Fife, UK; 3 School of Medicine, Fife Community Advisory Council, University of St Andrews, St Andrews, UK; 4 Edinburgh Clinical Trials Unit, Usher Institute, The University of Edinburgh, Edinburgh, UK

**Keywords:** Multimorbidity, Palliative Care, End of Life Care, Informal Caregiver, Family Caregiver, Qualitative, Patient and public involvement

## Abstract

**Background:**

Towards the end of life, people with multiple long-term conditions face an array of challenges associated with their growing healthcare needs including unclear illness trajectories and multifaceted uncertainty. Caregivers, most often family or friends, play a key role in providing and coordinating care at the end of life. This study aimed to explore, through the perspective of bereaved caregivers, the experiences of healthcare and support in the last year of life for people with multiple long-term health conditions.

**Methods:**

Interpretative Phenomenological Analysis was used to analyse semi-structured interviews with 12 bereaved caregivers who had cared for relatives with multiple long-term conditions over the last year of life. Public advisors with lived experience as caregivers were key contributors to this study, aiding in study conceptualisation, design and analysis.

**Results:**

There were five key group experiential themes identified: (i) Multiple conditions mixed in together impacting prognostication, (ii) Dynamic, coordinated care from healthcare services supported caregivers, (iii) Information from various sources helped caregivers make person-centred decisions, (iv) Caregivers had to decide when and where to seek help, with a preference for known, trusted healthcare professionals and (v) Being a caregiver has a changing, persisting impact.

**Conclusions:**

Bereaved caregivers faced challenges in appraising the multiple conditions that their relative had towards the end of life. This, alongside prior healthcare experiences and information gathered by caregivers, influenced how they accessed healthcare and support for their relative. Empowering caregivers and integrating their experiences into clinical practice will be key to supporting them in providing necessary care for those close to them at the end of their life.

**Supplementary Information:**

The online version contains supplementary material available at 10.1186/s12904-026-02115-y.

## Introduction

The number of people living with, and accordingly dying with and from, multiple long-term conditions (MLTCs), also known as multimorbidity – defined as the presence of two or more long-term conditions – is rising [1, 2]. Extra years living with MLTCs are not always spent in good health, with an evident association between the presence of MLTCs and declining quality of life [3]. With increased specialisation of healthcare services, people living with MLTCs are often under review by multiple specialties and teams, often for overlapping issues and with associated regular planned and unplanned healthcare utilisation [4, 5]. This situation of multiple healthcare teams being involved with a single patient can contribute to fragmentation of care and a lack of coordination [6, 7]. Higher use of healthcare services carries high healthcare costs both to societies and individuals, even in universal healthcare systems [8, 9].

With increasingly strained healthcare services, much of the load of caring for people with MLTCs relies on unpaid/informal caregivers, usually family members or friends. It is recognised that with increasing prevalence of MLTCs, caregivers may themselves have their own long-term conditions, impacting their ability to provide care [10]. Caregivers’ health has been shown to be impacted negatively by their caring roles, with reduced health related quality of life and increase in a range of physical and mental health conditions [10, 11]. Caregivers are often required to provide care on a daily basis, managing significant uncertainty about the conditions their dependent person is living with, navigating complex health and support systems and monitoring continual changes in needs, particularly towards the end of life [12]. Many caregivers caring for people with MLTCs at the end of life describe feeling undervalued by healthcare professionals, a situation that can be exacerbated by a lack of coordination and continuity of care [13].

As people approach the end of life, they often prefer to have the majority of their care outside of acute hospital settings [14] although we observe that their use of healthcare services particularly unscheduled, or unplanned, healthcare also increases at this time [15, 16]. Healthcare costs at the end of life remain predominantly directed to care provided in hospital settings [17] despite evidence of more people having end of life care in the community [18] and the fact that most people would choose less acute hospital care at the end of life [19].

Poor continuity of care in the community is known to trigger acute hospitalisation, along with distress in patients and their caregivers, rather than transfer to hospital being an explicit preference [20]. There is some evidence that having an awareness of prognosis can create opportunities for people to express their personal priorities and to shape decision-making about future care as they deteriorate [21]. Such future, or advance, care planning can be challenging within people with MLTCs whose illness trajectories are inherently unpredictable [22]. Critically, we do not know enough about the experiences of healthcare encounters in the months before death for people with MLTCs and their caregivers, and where any utility lies.

## Study aim

This study aimed to explore the experiences of healthcare and support in the last year of life for people with multiple long-term health conditions and those close to them through the perspective of their bereaved caregivers.

## Methods

### Theoretical framework

Interpretative Phenomenological Analysis (IPA) is a methodology suited for exploring sensitive, emotional, lived experiences of participants, ideally serving the objectives of this study [23]. The theoretical principles guiding IPA – hermeneutics, phenomenology and ideography – allow a detailed appreciation of each participant’s own account of that event [23].

### Participant selection

Participants were eligible for this study if they had provided care for someone:

- with two or more long-term conditions (as per a prior Delphi consensus [24]),
- who died between 3 months and 12 months ago (based on prior research [25] and the experiences of the PAG) and,
- both caregiver and decedents were aged ≥ 18 years.

Recruitment was focussed within one health board within NHS Scotland. Purposive sampling was utilised to ensure participants had a breadth of sociodemographic and clinical backgrounds – with particular consideration given to socioeconomic deprivation status, the types of long-term conditions and place of death. Data saturation is not a requirement of IPA, reflecting the individualised nature of personal, lived experiences [23].

A variety of pre-determined recruitment strategies were utilised to recruit bereaved caregivers. These included an initial focus on social media advertisements; allowing participants to self-identify via study-specific accounts on X, Facebook and LinkedIn. However, due to inundation by inauthentic participants [26, 27], this strategy was aborted. Recruitment was redirected through local third sector organisations, the Public Advisory Group (PAG) and clinical contacts within primary and secondary healthcare services. Potential participants then opted in via email. SPB screened potential participants, provided more study details and answered any questions prior to arranging an interview. Participants were offered a £25 shopping voucher as remuneration and thanks.

### Study setting

One-off face-to-face interviews between SPB and participants took place in participants’ own homes or in a university interview room, as decided by participants. Participants were invited to have a support person accompany them for the interview; however, data was not collected from them.

### Data collection

Demographic data were collected at the start of the interview. This was mainly self-described, although postcodes were used to record socioeconomic status via the Scottish Index of Multiple Deprivation (SIMD) 2020 [28]. Participants were assigned a pseudonymised code to allow linkage of demographics and the corresponding interview data.

A flexible semi-structured interview guide was used (Supplementary File 1). Interviews were recorded using a secure Dictaphone with audio files stored securely in the University of St Andrews OneDrive system. Interviews were then transcribed naturalistically by SPB to incorporate colloquialisms and mannerisms.

### Data analysis

The principles of Interpretive Phenomenological Analysis were used to guide iterative analysis of the transcripts as set out by Smith et al. [23]. SPB independently undertook stages 1–6 of analysis for each case using NVivo 14 [29]. EK and EM reviewed three different transcripts each to ensure congruence within the analysis and then with SPB, they collectively developed the Group Experiential Themes. These were further developed by the PAG in a co-analysis workshop, as detailed below.

### Ethical considerations

Ethical approval was obtained from the North of Scotland NHS Research Ethics Committee (reference 23/NS/0142) and the University of St Andrews School of Medicine Research Ethics Committee (reference MD17573).

Consideration was taken in the study design and interviews to ensure that participants were as comfortable as was possible and that the emotional impact of revisiting their experiences was handled sensitively. Distress protocols were developed for both participants and the interviewer to handle emergent distress appropriately (Supplementary Files 2 and 3). Time was taken at the start of each interview, prior to recording, to explain the study, answer any questions and obtain written consent from each participant.

### Rigour and reflexivity

The study has been reported in accordance with the COnsolidated criteria for REporting Qualitative research (COREQ) checklist [30].

The primary researcher, SPB, is a Palliative Medicine and General Internal Medicine resident doctor who undertook this research during her PhD. The remaining author team provided support and guidance through research design and analysis, guided by their clinical, personal and research experience.

SPB kept a reflexive diary throughout, acknowledging initial field notes and emerging thoughts or assumptions that may later affect analysis. Engaging EK and EM in development of the Group Experiential Themes and then the PAG in refinement of such themes minimised potential bias.

### Patient and public involvement

The PAG consisted of members with lived experience of caring for people at the end of life. They influenced the study design, developed the participant-facing material and guided choices around recruitment methods. The term caregiver was suggested by the PAG who approved the definition as “someone who provided physical, emotional, practical, financial or spiritual support to someone who needed help with their day to day needs and/or welfare”, in preference to other options including ‘carer’. A co-analysis workshop was held after the initial generation of Personal Experiential Themes to integrate public advisors’ lived expertise with the analysis of this study. During this workshop, public advisors reviewed the preliminary Personal Experiential Themes and their organisation into Group Experiential Themes. Their role was to provide insight, based on their own lived experience, to challenge or affirm the researchers’ interpretations, refine theme generation and nomenclature, and to identify quotations that best represented the experiential content of the themes. Members of the PAG are co-authors on this manuscript, ensuring that the study retains a focus on what matters most to people with multiple long-term conditions and their caregivers.

## Results

Twelve bereaved caregivers completed interviews, all of whom had cared for a family member (Table 1). Most participants were recruited via clinical contacts. Interviews lasted between 60 and 220 min. At the time of interview, the decedents had died in the preceding 3 to 13 months and all had multiple long-term conditions, with variation in the types of conditions reported (Table 2).

**Table 1:** Self reported bereaved caregiver characteristics, ^a^ Socioeconomic quintile measured by Scottish Index of Multiple Deprivation 2020 (1 is the most deprived quintile, 5 is the least deprived quintile)

Interview identifier	Gender	Age	Ethnicity	Socioeconomic quintile^a^	Caregiver relationship to decedent	Method of recruitment
P1	Female	30s	Mixed	2	Daughter	Clinical
P2	Female	80s	White	5	Wife	Clinical
P3	Female	50s	White	4	Partner	Clinical
P4	Female	60s	White	2	Wife	Clinical
P5	Female	60s	White	2	Daughter	Third sector
P6	Male	50s	White	2	Son	Public advisor
P7	Male	80s	White	4	Husband	Clinical
P8	Female	50s	White	1	Daughter	Clinical
P9	Male	60s	White	2	Son	Clinical
P10	Female	60s	White	1	Sister	Clinical
P11	Male	20s	White	4	Son	Clinical
P12	Female	40s	White	3	Daughter	Clinical

**Table 2:** Decedent characteristics, as reported by bereaved caregiver

Interview identifier	Gender	Age	Ethnicity	Caregiver reported health conditions	Place of death	Time since death at interview
P1	Female	50s	White	Chronic liver disease, Solid organ cancer, Haematological cancer	Hospice	4 months
P2	Male	80s	White	Solid organ cancer, Parkinsons disease, Stroke	Hospital	4 months
P3	Male	50s	White	Solid organ cancer, Asthma	Home	5 months
P4	Male	60s	White	Haematological cancer, Coronary artery disease, Hypertension, Osteoarthritis	Hospice	5 months
P5	Female	80s	White	Arrhythmia, Heart failure, Dementia, Osteoarthritis, Hypertension	Hospital	13 months
P6	Female	80s	White	Dementia, Chronic obstructive pulmonary disease, Pancreatic disease, Osteoporosis	Hospital	4 months
P7	Female	80s	White	Dementia, Stroke, Hypertension, Asthma	Hospital	11 months
P8	Female	70s	White	Dementia, Chronic obstructive pulmonary disease, Osteoporosis, Osteoarthritis, Heart failure	Care home	3 months
P9	Male	80s	White	Chronic kidney disease, Diabetes	Hospital	9 months
P10	Female	60s	White	Chronic kidney disease, Epilepsy, Congenital disease	Home	6 months
P11	Male	60s	White	Long-term musculoskeletal problems due to injury, Chronic pain, Peripheral arterial disease, Chronic obstructive pulmonary disease	Home	8 months
P12	Male	70s	White	Pulmonary fibrosis, Previous cancer (in remission), Hypertension	Home	8 months

Table 3. Provides an overview of the themes and sub-themes underpinning the findings. 

**Table 3:** Overview of Group Experiential Themes and sub-themes derived from Interpretative Phenomenological Analysis

Group Experiential Theme	Theme	Sub-themes
1	Multiple conditions mixed in together impacting prognostication	Conditions mixed together, impacting health
		Conditions aided prognostication but discussions were vague
		Caregivers were observant to deteriorations
		Had been there before
2	Dynamic, coordinated care from healthcare services supported caregivers	Knowing where to turn and feeling valued
		Having choice in healthcare decisions
		Coordination between healthcare services smoothed processes
3	Information from various sources helped caregivers make person-centred decisions	Knowing their relative as a person and their priorities
		Previous experiences impacted decisions made
		Sources of information gathered by caregivers
4	Caregivers had to decide when and where to seek help, with a preference for known, trusted healthcare professionals	Knowing when and how to access support
		Preferring trusted healthcare professionals
5	Being a caregiver has a changing, persisting impact	Caregiving wasn’t a choice, but it changed their life
		Coping with the impact of caregiving
		Ongoing impact

## Group experiential theme 1 – multiple conditions mixed in together impacting prognostication

Caregivers described challenges in differentiating between their relative’s various conditions. The interplay of conditions not only impacted their relative’s health and symptom experience but also prognostic awareness. Whilst caregivers reflected that they often recognised signs of deterioration in their relative, discussions with healthcare professionals about this subject were often vague and unclear.

### Conditions mixed together, impacting health

The caregivers described difficulties in knowing which conditions were causing which symptoms. Particularly, interactions were described between physical and mental health conditions which caregivers had to learn to appraise.

*And the learning process for me*,* I think was as much*,* it was a mix of the physical aspects and the mental aspects – P7*.

These various conditions were also seen to contribute to their relatives’ declining health. P8 describes her mother’s conditions contributing to an acute presentation.

*I got a second phone call and it just explained it a wee bit more wae [with] heart failure*,* wae [with] things like that because they are*,* and then with her having the COPD and her lungs being quite bad as well – P8*.

Caregivers also described how conditions impacted on decisions made by healthcare professionals, particularly around types of treatments available to them.

*Physio wouldn’t do anything*,* they wanted to do I think it was hydrotherapy*,* but they couldn’t do that until his hernia was sorted so he was in this vicious circle – P4*.

### Conditions aided prognostication but discussions were vague

For some, an understanding of these interacting conditions resulted in them knowing their relative would not survive. For P5, her mother’s various conditions and the resulting impact of these aided her understanding regarding limited treatment options.

*And he said that “there isn’t anything we can do other than to give antibiotics because your mum would not tolerate*,* you know*,* we couldn’t do an operation*,* she isn’t well enough and strong enough to survive an anaesthetic*,* so that would be our concern”. So*,* ok*,* that’s how it is*,* you know*,* so and and already at this point I was thinking whatever this is*,* mum’s not gonna [going to] get better” – P5*.

Other caregivers described that the diagnosis of particular conditions commonly assumed to be terminal such as cancer, made them aware of their relative’s limited prognosis.

*You know*,* if you’ve got cancer in your throat*,* you’re not going to survive much longer than a year or two anyway – P2*.

Conversely, absence of recognisably terminal conditions led to reduced prognostic awareness for caregivers and healthcare professionals.

*I think the difficulty for me was that nobody*,* it’s not*,* it wasn’t like cancer that somebody could say well you know what he’s only got six weeks*,* he’s only got a month. They couldn’t even say that he was end of life – P12*.

When prognosis was discussed by healthcare professionals, this was often vague involving implicit language rather than clear communication around anticipated timeframes. Again, the presence of particular conditions meant some healthcare professionals expressed uncertainty about prognosis, which P9 describes in a conversation with his father’s renal supportive care nurse.

*All she really said was and she just kept going with the fact that “your kidneys are sitting at that but they could stay like that for years to come” – P9*.

### Caregivers were observant to deteriorations

Even if discussions were not explicit regarding prognosis, caregivers were aware of their relative’s declining health. Occasionally, it was signs of their relative no longer being able to self-manage their long-term conditions that triggered awareness of their deterioration, particularly towards the end of life.

*It was very obvious he couldn’t take his asthma drugs. He couldn’t drink*,* certainly wasn’t eating. So we knew*,* we knew. – P3*.

From their close relationship with their relatives, and direct involvement in their care, caregivers often picked up on subtle signs of deterioration.

*I wouldn’t say she had a big thing*,* but all of the things that she had were becoming more frequent*,* more impactful on her overall well-being – P5*.

### Had been there before

However, due to the nature of their long-term conditions, many had experienced similar deteriorations previously. This could impact participants’ acceptance of deteriorations.

*No*,* no*,* it wasn’t expected because he did look better on the Tuesday than he had on previous occasions when we’d been in. He wasn’t looking great*,* but we’d seen him look worse. I didn’t think that was it – P9*.

For some though, surviving deteriorations before made them aware of the risk of these, aiding their understanding of the potential of subsequent declines.

*Then they said that she had a chest infection and I know when she recovered from that first one they told me “she’s recovered this time*,* but if your mum was to ever get a second one*,* there’s no*,* she would not beat a second time round”. So I was just absolutely devastated when they said it was another chest infection – P8*.

## Group experiential theme 2 – dynamic, coordinated care from healthcare services supported caregivers

Many participants shared that being supported by healthcare services who responded to their needs in a dynamic way resulted in positive experiences, described as a proactive, timely, flexible approach that met the emerging needs of their relative and allowed for choice. When participants did not feel listened to or supported, their experiences were less satisfactory.

### Knowing where to turn and feeling valued

Proactive healthcare team involvement was important to caregivers and aided their understanding about the roles and remit of that service. This empowered caregivers, enabling them to actively reach out to services when required and inspiring confidence.

*I was told I could ring up at any time*,* you know*,* to ask to speak to the nurse*,* which I did sometimes. Not just the regular phone call*,* but very often I would phone up about certain aspects*,* you know*,* what should I do? Should I do this? Would it be better to do that? – XA02*.

*the GP*,* as I say*,* we’ve got em GPs*,* they were out every other week touching base*,* and if they didn’t come out*,* if they were too busy*,* they would phone just making sure everything was okay – P12*.

Contrastingly, negative experiences were described when caregivers did not feel valued. P1 described feeling more listened to by the hospice inpatient unit compared to a prior hospital team.

*If you’ve got any questions*,* there’s always just somebody about*,* whereas compared to the hospital*,* they just couldn’t give a flying fuck about you – P1*.

Similarly, P12 expressed frustrations that her mother’s care home didn’t listen to her concerns about her mother’s declining health in her final weeks of life resulting in distress.

*It was horrendous like and I still feel that the care home could have done more for her and that was*,* that was the bit that really distressed me for the last four weeks of her life – P12*.

P1 described how a lack of support from healthcare professionals gave both her and her mother false reassurance that her condition was stable, feeding into their prognostic uncertainty.

*Because nobody was seeing anything or doing anything or giving us any information I was like “they’re probably not worried about it Mum” and I’m like “you’re probably*,* yeah*,* might have ages” – P1*.

Caregivers could feel so overwhelmed by the tasks in hand that they felt unable to determine themselves what help was needed.

*You’re too focussed on dealing with what you’re dealing with*,* you*,* you know*,* you don’t really have time for taking support from anybody else really – P12*.

### Having choice in healthcare decisions

Caregivers often preferred more active involvement in decision-making processes. For P6, having a mutually agreed time to receive updates about his mother’s care in hospital led to a positive encounter.

*They actually you know*,* I cannae [cannot] praise them enough*,* they were brilliant. You know*,* and I says [said] “look there’s no point in me phoning you on a Monday or a Tuesday for a weekly update at the beginning of the week.” I says [said] “what about I phone you on a Friday?” They says [said] “that would be great” – P6*.

Having such a personalised and flexible approach to care was experienced very positively. For P3, a bucket list, dream trip for her partner was made possible by the district nursing service refilling his syringe driver early.

*The nurses were brilliant because we had to arrange that his syringe driver would be changed twice in one day so that you could have that time the next day – P3*.

Where shared decision-making was not experienced, this not only impacted negatively upon perceptions of that care episode but could have a longer-lasting impact about the types of care people were willing to accept. P11 described his father having been pushed towards accepting a period of hospital admission by his specialist team; a decision which he later felt less certain of and which led to his father wanting to avoid future admissions.

*That three day spell in hospital was really*,* it it contributed to him*,* it contributed to his mobility deteriorating. And that was the last time he ever went into a hospital. There were a few more appointments they wanted after that we kept*,* we we were still getting letters in June about*,* you know*,* come into hospital and get this looked at or we want to look at this or whatever. And my dad*,* just my dad said to the district nurses “I’m not doing it” because his experiences with hospitals over the last year had just been really bad – P11*.

### Coordination between healthcare services smoothed processes

Caregivers described that continuity in care gave them confidence that there was oversight and coordination between various teams.

*When the palliative care doctors got involved*,* things immediately picked up and life did feel a bit easier. And we felt like we*,* we’d had a few battles removed that we didn’t have to deal with anymore – P11*.

Caregivers also described how care coordination ensured all involved healthcare professionals were kept updated, supporting their confidence in healthcare services.

*I presume they have conversations about these things and I mean… [GP] used to say everybody knows about [her husband] – P2*.

## Group experiential theme 3 – information from various sources helped caregivers make person-centred decisions

Caregivers often had to make decisions on their relatives’ behalf and advocate for them, particularly when it came to admission to hospital. Such decisions were aided by gathering information to enable decision-making aligned with their relatives’ known priorities.

### Knowing their relative as a person and their priorities

Having a close relationship with their relative, and sharing similar values, enabled caregivers to feel confident in making person-centred decisions in agreement with their relatives’ priorities and values.

*I think it was really about knowing [her partner] so well and knowing what he wanted and also thinking what would I want in that position as well – P3*.

Having such close involvement in their relative’s care meant they were able to recognise when their health changed and empowered them to advocate for action to be taken, sometimes urgently.

*They would get doctors in but it was myself that had to because I would say to the care home “my mum’s not right the day” – P8*.

Some caregivers described having open conversations with their relative allowing them to understand their priorities and preferences around care. However, for others such conversations did not happen either due to the opportunity not arising or that not being their communication style as a family. In such situations, healthcare professionals guiding discussions enabled preferences to be communicated.

*I mean he never discussed it with us*,* it was only like when [renal supportive care nurse] asked him. He didn’t gather us round and say this is what I want*,* it was just*,* that wouldn’t of happened in our family – P9*.

### Previous experiences impacted decisions made

Similarly to the aforementioned quote in Theme 2.2 from P11, many participants described negative experiences of care in hospital settings, often whilst managing other long-term conditions.

*Then she had her heart*,* atrial fibrillation diagnosis. She’d been in hospital for about a week having the treatment to try and bring it under control and so on. She was very frightened of hospitals*,* which was why she was in such a stramash [uproar]*,* you know*,* when she went in – P5*.

By understanding this, caregivers were able to advocate for their relative to key healthcare professionals. P2 recalls a conversation with her GP requesting a local community hospital for care, rather than a more distant tertiary hospital due to her husband’s prior negative experience there.

*Just saying “please*,* the one*,* the one thing that he has requested is please*,* please*,* if you can possibly do it” I mean you can’t obviously dictate but if it’s possible end of life*,* could we*,* you know*,* if he needs hospitalised could it be in [local hospital] – P2*.

### Sources of information gathered by caregivers

Caregivers sought out information from various sources. For some, their professional role provided both knowledge and acceptance of their relatives’ deteriorating health, such as P5 who worked in a role supporting carers.

*I just*,* I’m kind of a pragmatic person and I really*,* you know*,* people have their time and*,* and it’s one of the*,* you know*,* one of the things with*,* you know*,* working with carers – P5*.

Others spoke to personal contacts who held professional roles meaning that they could support sense-checking and interpreting information given to the caregiver by their relative’s healthcare professionals. P3 describes a friend who worked in the medical profession as being a useful contact to facilitate her conversations with healthcare professionals.

*When [her partner] was on steroids*,* I’d phoned [friend] at some point and said “Look*,* should*,* this is happening. Do you think*,* it’s*,* should I be asking them to increase the steroids and*,* or is there any point in waiting?” And he told me exactly how he felt and then I was able to talk to his specialist nurse and doctors about. So it was really good. – P3*.

Many of the caregivers also described online resources equipping them with the knowledge to understand what was happening and prepare them for supporting their relative, enabling them to feel prepared and gain an understanding of prognosis.

*All the symptoms that she was going through was what I read. So I felt that helped a bit so I knew what to look for and what stages we were at – P10*.

Others with regular input from healthcare professionals knew they could rely on them to answer their queries and concerns.

*But at every point that the district nurses were coming*,* if there was any question that I had*,* I was able to ask them – P3*.

## Group experiential theme 4 – caregivers had to decide when and where to seek help, with a preference for known, trusted healthcare professionals

Caregivers described their decision-making around accessing healthcare, often driven by emergent symptoms or other unmet needs. There was a clear desire to access healthcare professionals with whom they had pre-existing relationships.

### Knowing when and how to access support

Many of the caregivers describe being able to access the required support when necessary. However, some felt that the processes required to access some services, such as the NHS24 telephone advice line, were lengthy and did not align with their relative’s health needs, particularly when urgent care was necessary.

*So anyway I phoned the NHS24 (sighs). So I don’t really need to go through all that crap but uh for fuck sake 40 min of asking questions*,* she could be deid [dead] in 20.* – P6.

Caregivers took on responsibility for monitoring and appraising their relative’s health and some were able to utilise this information to guide decision-making about which healthcare service to contact.

*If he wasn’t feeling well*,* if it’s something I felt like when he got the wheezes I could tell so yeah*,* that’s the doctor (GP). It was “Can you come out and see him?” “Aye we’ll be oot [out]”. If it was anything worse like he was short of breath*,* or there was one time he had a pain down his side so that was 911 (999 – ambulance service) – P9*.

However, decision making could be challenging and other caregivers toiled with knowing if it was necessary to contact services and the optimal timing for this. It was evident that such decisions became clearer with the experience of being a caregiver.

*Sometimes making the decision*,* that decision*,* is this the time to phone? Should*,* is this something that I can cope with*,* you know*,* or is should I get some help? And yeah*,* you know that obviously latterly it became more obvious when I needed help. But to begin with it was a bit difficult*,* you know. – P2*.

### Preferring trusted healthcare professionals

Caregivers preferred continuity in the individual health and social care professionals they encountered. They built up relationships with these healthcare professionals and this aided their trust in them.

*They’ve got like three carers that I was close to*,* to look after her. So I went home on that first night on the Saturday – P8*.

There were times when caregivers would rather wait until such trusted health and social care professionals were back on shift, rather than utilise emergency or unscheduled care services.

*So we were up all night and I thought I’ll wait till 8:00 and then the local nurses would be on shift then and I phoned and it was one of the nurses that came in quite regular. – P10*.

The consequences of not having continuity could be problematic and lead to negative experiences. P11 describes a contrasting experience to the specialist palliative care team who he got to know very well, with the GPs at his local health centre.

*We were also dealing with doctors from (GP Practice) health centre at that time. Our experience with them wasn’t as seamless and as*,* as good. Part of that is down to the fact that we never dealt with the same person – P11*.

## Group experiential theme 5 – being a caregiver has a changing, persisting impact

Caregiving wasn’t always a choice for participants, and they had to find ways to cope with the impact of this, which could be lasting.

### Caregiving wasn’t a choice, but it changed their life

Many of the caregivers described a sense of duty in their role, seeing this as the only option to allow their relative to receive the care they required and desired.

*It was caring for my father but I never looked at it as caring. It was always just a case of this is how it is. We do what needs to be done – P11*.

Caregivers also described feeling that their relative would have done the same for them as a key motivator.

*He was a very*,* very caring person. So it was nice to sort of do it for him too*,* you know. And I know he’d have done the same for me. Show him*,* that’s*,* you know*,* that’s what partnership is about*,* isn’t it? Take the good and the bad. – P2.*

However, many described putting their lives on hold in order to be there and support their relative.

*I stopped living*,* I was just existing*,* and my life was dedicated to them – P8*.

### Coping with the impact of caregiving

There were wider impacts experienced by caregivers. The physical impact of caregiving was described with interrupted sleep patterns and consequences for caregivers’ own health, as they prioritised caring for their relative. This could subsequently influence decisions around using healthcare, such as when P4’s husband was admitted to the hospice.

*I was exhausted*,* I was absolutely exhausted. And she just said “Do you think a stay in the hospice would help?” – P4*.

There were also wider emotional impacts of caring, which interfered with caregivers perceived ability to carry out their role. P6 describes the stress associated with managing his mother’s medications.

*There were times when I was writing down when she was taking them coz there was times where I was forgetting. I was just so stressed – P6*.

Despite these impacts, caregivers often found ways to cope to provide the necessary care their relative required.

*I was getting like that coz I was so frustrated and it was affecting my health and as I said to you*,* I mean if I go down*,* mum’s gone*,* they’ll take her away – P6*.

### Ongoing impact

The impacts of caregiving were described by some as lasting beyond the death of their relative. Many of the caregivers describe persisting guilt and rumination over decisions they made, particularly around seeking help from healthcare professionals.

*Could I have picked up that phone sooner than that Sunday night into the Monday morning when he was struggling to breathe and I was trying to sleep and if I had maybe phoned an hour before (pauses) would things be different? – P12*.

Many of the caregivers also experienced the consequences of putting their lives on hold at this time. P4 describes her husband’s death as leaving a void in her life.

*And then afterwards you’re left with this big hole really*,* because you’re no longer caring because even when he was in the hospital*,* I was still looking after him*,* I was still caring for him – P4*.

For P11, becoming his father’s caregiver at a crucial point in his life, as he was completing University, is impacting his life very significantly.

*I’m essentially*,* I’m going to have to start building a life for myself this year*,* which I haven’t been able to. I mean*,* I’m essentially*,* I’m 10 years behind everyone else my age – P11*.

For many though, the opportunity to be a caregiver, although difficult, was seen as something they are glad they were able to do.

*I really did think it was an absolute privilege to be able to look after him. You know*,* he could have picked anyone and he picked me – P3*.

## Discussion

This study highlights the complex and often under-recognised experiences of caregivers supporting people towards the end of life. Understanding these experiences is important not only for supporting individual caregivers, but also for informing the design of services and policies to improve care delivery and experiences at the end of life. The role of the caregiver during this phase of life emerged as pivotal, with caregivers fulfilling a great variety of essential functions.

The various conditions of decedents mixed together, which was not only a learning experience for caregivers but added uncertainty to interactions with healthcare professionals. Some participants cared for people with more obviously terminal or advanced conditions, such as cancer, where their (and perhaps their healthcare professionals’) understanding of the expected trajectory was clearer. Where this awareness was less apparent, conversations regarding prognosis with healthcare professionals, patients and caregivers were vaguer, fuelling the uncertainty experienced by these caregivers and others [12]. This frustrated caregiver participants, who described the lack of acknowledgement of prognostic uncertainty having negative consequences. Recent research has advocated for more honest conversations, even where uncertainty is the reality [31]. The experiences noted, highlight that honest conversations with healthcare professionals may have reduced the negative impact of uncertainty on our caregiver participants and perhaps improved their and their relatives’ care experiences.

There was however limited evidence that prognostic awareness, or the lack of this, impacted decision-making about healthcare. Other factors appeared to influence this more – particularly previous experiences with healthcare services, knowing their relative and their priorities, and the information that caregivers obtained. Caregivers had to be resourceful, particularly those without proactive healthcare teams supporting them. Many utilised their own prior professional knowledge and personal contacts to help them, often in place of contacting healthcare professionals. Some caregivers had access to services, such as palliative care advice lines, that were available to support their evolving needs, though others relied on gaining their own information, particularly from the internet, through websites they deemed as trustworthy. Utilising such technologies as resources can promote caregiver empowerment and capacity, but barriers can exist in accessing these, and consideration must be given to the accuracy of online health information [32]. Therefore, consideration must be given to levelling-up the information available to all people approaching the end of life, and their caregivers.

One of the most consistent findings in this study was the connection between trusted relationships with healthcare professionals, the ability to navigate healthcare services and resultant experiences of these interactions. Such relational continuity is a well-recognised strategy for improving care for people with MLTCs, with much of the work focussed on GPs [33, 34]. Beyond GPs, participants in this present study spoke of other healthcare professionals, such as district nurses, haematologists, renal nurses and specialist palliative care staff, as being key providers they built relationships with and trusted. Trust impacted decisions about accessing healthcare, with some participants describing a preference to wait for certain individuals or services to be available rather than utilising unfamiliar services. In addition to relational continuity, informational continuity (sharing of information between healthcare teams) was important to caregivers. These types of continuity have been shown to impact outcomes and satisfaction with palliative care [35]. Continuity is therefore an essential consideration for future models of healthcare delivery, particularly exploring which of the various services involved in the care of people with MLTCs are best placed to provide this.

Caregivers often felt compelled to undertake this role, putting their lives on hold to strive to meet their relatives’ needs. Being a caregiver had a persistent impact on their lives, with many describing wide-reaching consequences. Previous literature has shown that caring is associated with impacts on caregivers’ physical and mental health, directly correlating with the intensity of care provided [36]. Many participants in this study described impacts on their employment, either through short-term absences or longer-term loss of role. Most prior research into employment consequences has focussed on the impact whilst directly providing care [37] although there have been recent developments in upskilling employers to better support bereaved caregivers [38]. These findings underscore the importance of tailored support for caregivers, which can improve both caregiver well-being and patient experiences of care.

### Strengths & limitations

This study created the opportunity for caregivers to harness their experiences to improve understanding around healthcare decision-making and experiences for people with MLTCs and those close to them. Every step of this study has been undertaken in collaboration with a team of public advisors, each with their own lived experiences. This has strengthened the study and ensured it was carried out sensitively to comprehensively capture the breadth of experiences that come with being a caregiver of someone at the end of life. Involving the public advisors and other researchers in the analysis of this study, ensured bias was minimised and further strengthened the work.

There was much consideration and planning to maximise the diversity of participants involved in this study. Participants came from a variety of socioeconomic backgrounds and the people they cared for had differing long-term conditions. There were more females than males who participated, likely reflecting the demographic of those who provide such care. One limitation to this work is that it is set within a specific region of Scotland, which may limit its transferability to other settings. Despite attempts to diversify this study, almost all participants were of white ethnicity, which is a further consideration for generalisability. Most participants were recruited via clinicians, which may have resulted in over-representation of caregivers who were already engaged with healthcare services. As a result, the experiences of caregivers who were less connected with services, or who faced barriers to accessing care, may be under-represented – though it is noted a variety of experiences were evident, regardless of recruitment pathway.

## Conclusion

This research highlights, through robust methodology and strong public involvement, that caregivers supporting people with MLTCs play a pivotal role in navigating complex healthcare systems towards the end of life. Their contributions are critical to the quality of care received, yet the enduring physical, emotional and practical consequences of this on caregivers are underappreciated. These findings highlight the need for health and social care systems to recognise and support caregivers as integral partners in care. Future work should actively involve caregivers in the development of adaptive, person-centred health and social care models, ensuring these address not only the needs of people nearing the end of life but also prioritise caregivers’ own role, wellbeing and resilience.

## Supplementary Information

Supplementary Material 1.

Supplementary Material 2

Supplementary Material 3.

## Data Availability

Access to pseudonymised transcriptions for secondary data analysis will be considered with reasonable requests via the corresponding author.
