# Supplementary material for: “We do what needs to be done”: caregivers’ experiences of healthcare and support for people with multiple long-term conditions in the last year of life
Source: BMC Palliat Care. 2026 May 27;25:215. doi: 10.1186/s12904-026-02115-y (PMC13393848; doi:10.1186/s12904-026-02115-y)
Supplement: Supplementary file 1 — Supplementary Material 1. [file 12904_2026_2115_MOESM1_ESM.docx]

| **Main Question** | **Prompts/Follow Up Questions** |
| --- | --- |
| *Opening question* | |
| I wonder if we could start by you telling me a bit about [decedent’s name] |  |
| What was your relationship with [decedent's name]? | Did you live nearby? |
|  | Did this change at all during their life? |
| *Last year of life* | |
| Could you tell me what [decedent’s name]’s last year of life was like? | Where were they living, who was around, what did they do day to day? |
|  | What role did you have during that time?” |
| What was important in that last year of life? To them and to you? | Did they prioritise where they were? |
|  | Was their health on their mind? |
| How did their different health conditions impact what happened in that last year? | Do you think [decedent’s name] achieved what was important to them in that last year? |
| *Healthcare* | |
| Did [decedent’s name] have any healthcare interactions in the last year of life? | That might include things like visits to the GP or hospital or district nurses or dentist or other clinics |
|  | What were their experiences at these interactions? |
| What prompted these healthcare appointments? | Did they focus on one condition or address lots of condition? |
|  | Did these appointments meet their needs? |
| Did the care feel personal/individual to [decedent’s name]? |  |
| Did you receive any specific help or support as a caregiver? | From family/friends or from professionals? |
| *Prognostic awareness* | |
| At what point in the last year of [decedent’s name]’s life did you realise that they may be dying? | Was this ever discussed with anyone else? |
|  | Did anything change after you had this information, and if so in what way? |
| Did any of [decedent’s name]’s particular health conditions particularly prompt discussions that they may be approaching the end of their life?” |  |
| Was [decedent’s name] given an opportunity to plan or have their say in how they may be cared for in the last year of life? |  |
| *End of Life* | |
| Would it be ok for you to tell me what the time leading up to [decedent’s name]’s death was like? | Where were they cared for? |
|  | Did you or they receive any specific professional support e.g. carers/district nurses? |
| When did you realise that [decedent’s name] was dying? | Did any healthcare professionals tell you that [decedent’s name] might be dying? |
| What impact did [decedent’s name]’s death have on you? | Did you feel supported after [decedent’s name] death? |

Participant interview guide
