# Supplementary material for: “We do what needs to be done”: caregivers’ experiences of healthcare and support for people with multiple long-term conditions in the last year of life
Source: BMC Palliat Care. 2026 May 27;25:215. doi: 10.1186/s12904-026-02115-y (PMC13393848; doi:10.1186/s12904-026-02115-y)
Supplement: Supplementary file 2 — Supplementary Material 2 [file 12904_2026_2115_MOESM2_ESM.docx]

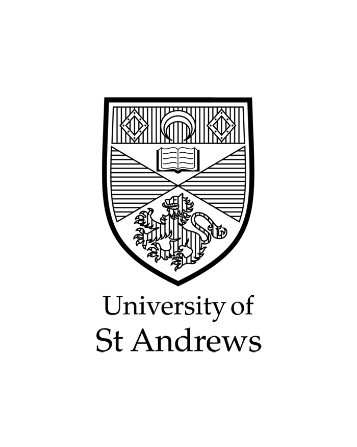
**Participant Distress Protocol**

**Experiences of healthcare and support for people with multiple long-term health conditions in the last year of life and their caregivers**

**EMBED** – **E**xperiences of **M**ultimor**b**idity at the **E**n**d** of Life

Dr Sarah Bowers, Dr Jo Bowden, Professor Frances Quirk

Participant Distress Protocol – modified from Wright et al(1), Draucker et al(2) and Whitney et al(3).

1. Wright N, Hadziosmanovic E, Dang M, Bales K, Brookes C, Jordan M, et al. Mental health recovery for survivors of modern slavery: grounded theory study protocol. BMJ Open. 2020;10(11):e038583.

2. Draucker CB, Martsolf DS, Poole C. Developing distress protocols for research on sensitive topics. Arch Psychiatr Nurs. 2009;23(5):343-50.

3. Whitney C, Evered JA. The Qualitative Research Distress Protocol: A Participant-Centered Tool for Navigating Distress During Data Collection. International Journal of Qualitative Methods. 2022;21:16094069221110317.
