# Supplementary material for: “We do what needs to be done”: caregivers’ experiences of healthcare and support for people with multiple long-term conditions in the last year of life
Source: BMC Palliat Care. 2026 May 27;25:215. doi: 10.1186/s12904-026-02115-y (PMC13393848; doi:10.1186/s12904-026-02115-y)
Supplement: Supplementary file 3 — Supplementary Material 3. [file 12904_2026_2115_MOESM3_ESM.docx]

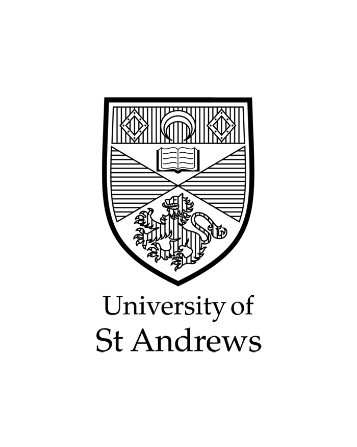


**Experiences of healthcare and support for people with multiple long-term health conditions in the last year of life and their caregivers**

**EMBED** – **E**xperiences of **M**ultimor**b**idity at the **E**n**d** of Life

Dr Sarah Bowers, Dr Jo Bowden, Professor Frances Quirk

**Researcher Distress Protocol**

Researcher Distress Protocol – modified from Wright et al(1).

1. Wright N, Hadziosmanovic E, Dang M, Bales K, Brookes C, Jordan M, et al. Mental health recovery for survivors of modern slavery: grounded theory study protocol. BMJ Open. 2020;10(11):e038583.
